# An instrument assessing patient satisfaction with day care in hospitals

**DOI:** 10.1186/1472-6963-12-125

**Published:** 2012-05-24

**Authors:** SM Kleefstra, RB Kool, LC Zandbelt, JCJM de Haes

**Affiliations:** 1 Kiwa Prismant, Research institute for health care, Department Quality and Safety, Utrecht, the Netherlands; 2 Scientific Institute for Quality of Healthcare, Radboud University Medical Centre Nijmegen, Nijmegen, the Netherlands; 3 Academic Medical Centre, Department Quality and Process Innovation, University of Amsterdam, Amsterdam, the Netherlands; 4 Academic Medical centre, Department Medical Psychology, University of Amsterdam, Amsterdam, the Netherlands

**Keywords:** Patient satisfaction, Day care, Hospitals, Improving quality of care

## Abstract

**Background:**

Patient satisfaction is an important indicator of quality of care in hospitals. Reliable and valid instruments to measure clinical and outpatient satisfaction already exist. Recently hospitals have increasingly provided day care, i.e., admitting patients for one day without an overnight stay. This article describes the adaption of the ‘Core questionnaire for the assessment of Patient Satisfaction’ (COPS) for general Day care (COPS-D), and the subsequent validation of the COPS-D.

**Methods:**

The clinical COPS was supplemented with items to cover two new dimensions: *Pre-admission visit* and *Operation Room.* It was sent to a sample of day care patients of five general Dutch hospitals to investigate dimensionality, acceptability, reliability, construct and external validity. Construct validity was established by correlating the dimensions of the COPS-D with patients’ overall satisfaction.

**Results:**

The COPS-D was returned by 3802 patients (response 46%). Factor analysis confirmed its’ structure: *Pre-intake visit, Admission, Operation room, Nursing care, Medical care, Information, Autonomy* and *Discharge and aftercare* (extraction communality 0.63-0.90). The internal consistency of the eight dimensions was good (α = 0.82-0.90); the item internal consistency corrected for overlap was satisfactory (>0.40); all inter-item correlations were higher than 0.45 but not too high (<0.90). The construct validity of all dimensions was good (r from 0.52-0.62, p < 0.01). The *Information* dimension had the strongest correlation with overall day care satisfaction.

**Conclusions:**

The COPS-D is a reliable and valid instrument for measuring satisfaction with day care. It complements the model of measuring patient satisfaction with clinical and outpatient care given in hospitals. It also fulfils the conditions made while developing the clinical and outpatient COPS: a short, core instrument to screen patient satisfaction.

## Background

In recent years hospitals have increasingly been providing day care, i.e. the admission of patients to a hospital during one day without an overnight stay. OECD figures show an increase of day care surgical procedures of 121 per cent in the period between 2000 and 2007 in seven European countries and Australia [1].

Substitution of clinical care by day care can have several consequences for the hospital. It often results in new centres with a different philosophy and logistics. For instance, hospitals can rationalise their inpatient bed utilization with reduction of admissions and intra-treatment transfers [2-4]. A study on technological innovations in surgery showed that as more patients were treated on an outpatient basis, fewer hospital beds were needed, and traditional operating rooms had to adapt to a greater turnover of patients. In addition, postoperative care is carried out in the community rather than in hospitals [2,4]. Also reduction of costs is mentioned as a result of substituting day care for clinical care [2-8].

Day care admissions may have advantages from the patients’ perspective such as prevention of hospitalization, decrease in waiting times and a more rapid recovery. Patients prefer to recover at home because it disturbs their lives minimally [5]. It is generally appreciated by patients that they can sleep at home and come to the clinic only a few hours before the procedure takes place [6]. Besides, different studies showed good clinical outcomes when clinical care was replaced by day care for totally different specialities like geriatrics, oncology, neurology, surgery and ophthalmology and the treatment of venous thrombosis or laparoscopic cholecystectomy [2,4-8]. Also day care may lead to fewer hospital-related infections [6]. Geriatric day hospitals for instance helped to avoid or shorten hospitalization and might contribute to the return of the patient to his home setting by facilitating the patients’ autonomy and the quality of life [8]. The availability of different medical and paramedical staff for frail elderly was a major advantage [7].

Day care treatment may also have disadvantages for patients. They may feel abandoned or unsafe or feel they are being sent home ‘too early’ [3,4]. Patients discharged within 24 hours after surgery might be at risk for early complications or readmission [3]. Parents of oncology patients reported inadequate information concerning appropriate home care and possible patient reactions, a lack of privacy and an increase of anxiety connected with having to take over too much responsibility [9]. Day care patients experience significantly higher levels of pre-operative stress and anxiety than do inpatients [6]. Another disadvantage mentioned in the literature is the number of examinations in geriatric hospitals in one day. This might be stressful and exhausting for elderly patients [8]. Finally, special attention is needed for patient-centred discharge procedures to prevent rehospitalisation [10]. Coping post discharge is a source of concern for patients, particularly if living alone. Patients need to be reassured and confident that they have access to further advice if required [5].

The experiences of patients with day care are therefore important when hospitals evaluate quality of their care [11,12]. In the Netherlands since 2004 almost all hospitals are using the same patient satisfaction questionnaire for clinical and outpatient settings, the so called Core Questionnaire for the assessment of Patient Satisfaction (COPS). This is a valid and reliable questionnaire for clinical and outpatient settings [13]. It provides benchmark information on a hospital as well as speciality level, including information on best practices. Reliability and validity of the COPS support the use of this questionnaire in assessing quality improvement interventions [14].

Over the last few years, more and more hospitals in the Netherlands indicated their need for a valid and reliable instrument to measure patient satisfaction with day care admissions. The existing patient satisfaction questionnaire COPS was not developed for measuring satisfaction of day care patients and lacked important issues relevant to this patient group.

After interviewing fifteen hospitals, including the eight academic hospitals, and a review of international literature, we concluded that, to the best of our knowledge, a valid questionnaire for day care satisfaction did not exist. Therefore, we decided to develop and validate a patient satisfaction questionnaire especially for day care admissions, based on the COPS. In this paper we describe the development and psychometric properties, i.e., the dimensionality of the COPS-Day care (COPS-D), by testing the acceptability, the reliability and the construct validity. To support construct validity we expect a moderately strong relation between the dimensions of the COPS-D and patients’ overall rate of satisfaction.

## Methods

### Instrument

We composed a questionnaire for day care patients, based on the COPS [13]. The COPS is a short core questionnaire to measure patient satisfaction, based on the needs of clinical patients and outpatients of academic hospitals. The questionnaire was developed to compare satisfaction scores between hospitals, and to identify opportunities for quality improvement.

The COPS consists of six dimensions, each dimension is covered by two, three or four questions: *Admission procedure* (3 items), *Nursing care* (2 items), *Medical care* (2 items), *Information* (4 items), *Autonomy* (3 items) and *Discharge and aftercare* (3 items).

For the COPS-D we included the six dimensions (17 items) of the COPS, assuming that these dimensions are as relevant to day care patients as they are to clinical and outpatients. In addition, we asked quality staff members of five general hospitals that had indicated a need for a day care patient satisfaction questionnaire to indicate which questions they found necessary to add in order to measure patient satisfaction in their day care organisation. Based on the needs formulated we added two new dimensions to the existing six dimensions from the COPS: *Operation room* (6 items) and *Pre-admission visit* (4 items). See Appendix 1 for the items used. In total the COPS-D consists of 27 questions. The same answering categories were used as in the COPS: a 5-point Likert-scale (1 = unsatisfied, 2 = somewhat satisfied, 3 = rather satisfied, 4 = quite satisfied and 5 = very satisfied). A dimension score is composed by adding the item scores and dividing the resulting total score by the number of items.

Besides the COPS-D, the questionnaire includes an overall rate for satisfaction with the patient’s treatment and stay in the hospital (range 0 unsatisfied to 10 very satisfied), and questions to assess patients’ background characteristics (i.e., age, gender, level of education) and a rating of perceived health status (bad, moderate, good, very good, excellent).

### Procedure

The COPS-D was tested in the five participating hospitals between November 2005 and May 2008 by sending a questionnaire to a sample of day care patients. We randomly selected 200 patients of each participating day care specialty who had visited the day care facility within the last six months. They received the questionnaire at home, accompanied by a letter from the hospital informing them about the questionnaire’s background. The questionnaire could be returned to an independent research institute in a pre-stamped envelope. A reminder was sent after two weeks. A helpdesk using phone and email was installed for patients needing support.

### Analyses

First we calculated the **correlations** of the newly added dimensions *Operation room* and *Pre-admission visit* with the original COPS-items. If these correlations were higher than 0.7, the items in the dimensions *Operation room* and *Pre-admission visit* might be measuring the same concept as the original dimensions in the COPS could be deleted without loss of information.

We tested the **construct validity** of the eight dimensions using a confirmatory principal component factor analysis with varimax rotation and eigenvalues greater than 1 [15]. We calculated extraction communalities as estimates of the variance in each variable accounted for by the factor. Small values indicate variables that do not fit well with the factor solution. The communality fits with a threshold of more than 0.4 [14]. We also investigated the Measures of Sampling Adequacy (MSA). This statistical analysis tests whether the sample fits the a priori defined model. If values are lower than 0.5 this may indicate that the variable does not seem to fit with the structure of the other variables.

Construct validity was also tested by calculating Spearman’s rank correlation coefficients of the eight dimensions with each other and with the overall satisfaction. A ρ-value ≥ 0.5 is considered to represent a strong correlation; 0.35 to 0.5 a moderate correlation; and 0.2 to 0.34 a weak correlation [16].

We tested the **reliability** of each dimension by calculating the Cronbach’s α. The α should preferably be higher than 0.7 [17,18]. In addition, we calculated the inter-item correlations and the item-total correlations (ITC) corrected for item overlap (item internal consistency). The inter-item correlations within a dimension should preferably be ≥ than 0.45. If the inter-item correlations are high (0.6 or 0.7), this indicates that 3 to 5 items will suffice in the dimension. If the inter-item correlations tend to be low (0.3 or 0.4), more items must be added to the dimension with a minimum of 7 to yield acceptable α’s [17]. Too high inter-item statistics (≥ 0.9) can indicate a redundancy of an item. Item-total statistics show the Cronbach’s α if an item is deleted: if this α is higher, the item should preferably be deleted. Also the item internal consistency should be larger than 0.4 [18]. We reported floor and ceiling effects to assess the skewness of the scores. The floor effect refers to the percentage of patients giving the worst possible score (namely 1 = unsatisfied). The ceiling effect refers to the percentage of patients giving the highest possible score (namely 5 = very satisfied).

Next, we tested the item discriminant validity (IDV). We correlated the items with the dimensions. The items should correlate more strongly with the dimension they are supposed to fit in than with the other dimensions [19].

Next, we checked the **external validity** of the sample used: whether the results can be extrapolated to the population of day care patients [20]. We compared the figures of the Dutch National Medical Registration (LMR) [21] on day care admissions in 2008 of the same eight specialties on gender and age with our sample. We expect that the results can be extrapolated based on our sample.

Furthermore, we tested **known group differences** using an ANOVA with a Bonferronni post hoc analysis. We expect elderly, lower educated and healthier patients to report higher overall satisfaction on all dimensions. We also expect that gender does not have a significant effect on all dimensions [12,22,23].

Finally, we tested the **acceptability** of the questionnaire by checking response rates and the missing values. The answering category ‘not applicable’ was excluded from the analyses. Items with a relatively high number of missing values (more than 10%) must be avoided [24,25] and might be left out of the basic questionnaire as they may not be applicable or relevant.

Data were analyzed used IBM SPSS 15.0.

## Results

### Sample

The COPS-D was sent to 8355 patients discharged from a day care unit from the five general hospitals. In total, 3802 patients returned and completed the questionnaire. The average response rate was 46% (range from 38% till 60%) See Table 1 for patient characteristics.

**Table 1:** Patient characteristics (n = 3802)*

**Item**	**Characteristic**	**N (Percentage)**
**Gender**	Female	2025 (53%)
	Male	1677 (44%)
**Age**	Younger than 20 years	234 (6%)
	20-59 years	1776 (47%)
	60 years or older	1754 (46%)
**Education**	Lower level	2647 (70%)
	Higher level	1013 (27%)
**Health status**	Bad/moderate	843 (22%)
	Good, very good, excellent	2412 (63%)
**Specialty**	Surgery	612 (16%)
	Internal Medicine	474 (13%)
	Orthopaedics	437 (12%)
	Gynaecology and Obstetrics	318 (9%)
	Ophthalmology	310 (8%)
	Ear Nose Throat-surgery	287 (8%)
	Cardiology	276 (7%)
	Urology	206 (6%)
	Other (less than 5% per specialty)	830 (22%)

* Numbers do not always add up to 3802, due to missing values.

### Dimensions *operation room* and *pre-admission visit*

Three items of the dimension *Operation room* correlated highly (> 0.7) with two items of the COPS, ‘Personal attention surgeon’ (correlation 0.742 with ‘Personal attention doctor’), Information surgeon’ (correlation 0.715 with ‘Personal attention doctor’) and ‘Transfer of information’ (correlation 0.708 with ‘Transfer of information’).

The correlations found in the dimension *Pre-admission visit* were all weaker than 0.627.

### Construct validity and reliability

The confirmatory factor analysis confirmed the structure of eight dimensions. 73% or more of the variance was explained by the dimensions (range 73,3% to 89,8%, mean 79%,) see Table 2. All items showed an extraction communality ≥ 0,45 (range 0,634 to 0,898). All items showed a MSA ≥ 0.5 (range 0,651 to 0,889).

**Table 2:** Factor analysis

**Dimension and items**	**N**	**% variance**	**Extraction**	**COPS or COPS-D**	**Measures of Sampling Adequacy (MSA)**
**Pre-admission visit**	**1243**	**77,9%**		**COPS-D**	
Reception	1243		0,739		0,861
Personal attention	1243		0,801		0,806
Expertise	1243		0,808		0,799
Information and instruction	1243		0,769		0,849
**Admission**	**1807**	**78,8%**		**COPS**	
Reception	1807		0,747		0,782
Rapidity of being able to speak to	1807		0,825		0,688
Degree of support	1807		0,790		0,724
**Operation Room**	**1307**	**81,2%**		**COPS-D**	
Reception	1307		0.813		0,726
Personal attention operation staff	1307		0.860		0,675
Expertise operation staff	1307		0.766		0,796
**Nursing care**	**3691**	**89,8%**		**COPS**	
Personal attention	3691		0.898		0,5
Expertise	3691		0.898		0,5
**Medical care**	**3355**	**89,7%**		**COPS**	
Personal attention	3355		0,897		0,5
Expertise	3355		0,897		0,5
**Information**	**3126**	**73,3%**		**COPS**	
Information by nurses	3126		0,730		0,845
Information by doctors	3126		0,783		0,805
Transfer of information	3126		0,785		0,806
Rapidity research results	3126		0,634		0,889
**Autonomy**	**1661**	**73,3%**		**COPS**	
Self-sufficient	1661		0,770		0,664
Participation in treatment decisions	1661		0,790		0,651
Privacy	1661		0,640		0,802
**Discharge**	**1248**	**78,4%**		**COPS**	
Information about further treatment	1248		0,810		0,704
Transfer of information to external professionals	1248		0,770		0,751
Discharge procedure	1248		0,772		0,748

Table 3 shows that good Cronbach’s α’s were found for the eight dimensions (range 0,816 to 0,906). The item internal consistency is supported by levels higher than the threshold of 0,40 (range 0,589 to 0,825). Also, all inter-item correlations were higher than 0,45 but not too high (<0,9) (range 0,511 to 0,797). The item internal consistency being high affirmed that the number of items in the dimensions is sufficient.

**Table 3:** Dimension characteristics of COPS-D: dimensions, mean, SD, Cronbach’s α, α if item deleted, item-internal consistency (ICC), item-discriminant validity (IDV), floor effect (floor), ceiling effect (ceiling)

**Dimensions**	**Mean ± SD**	**Cronbach’s α**	**α if item deleted**		**IIC**		**IDV**		**Floor (%)**	**Ceiling (%)**
			Min	Max	Min	Max	Min	Max		
Pre-admission visit	4,00 0,66	0,906	0,868	0,890	0,753	0,813	0,452	0,638	0,1	14,7
Admission	4,22 0,65	0,865	0,774	0,808	0,704	0,781	0,342	0,716	0,2	26,6
Operation Room	4,16 0,68	0,885	0,791	0,876	0,729	0,825	0,432	0,556	0,1	24,7
Nursing care	4,08 0,77	0,887	-	-	0,797	0,797	0,477	0,676	0,8	27,1
Medical care	4,07 0,85	0,882	-	-	0,793	0,793	0,412	0,7	0,8	30,4
Information	3,87 0,80	0,877	0,824	0,874	0,656	0,781	0,416	0,729	0,6	14,9
Autonomy	3,83 0,75	0,816#	0,692	0,827#	0,589	0,719	0,411	0,565	0,6	11,6
Discharge	3,72 0,86	0,861	0,781	0,820	0,724	0,765	0,370	0,713	1,0	13,9

# Cronbach’s α increases if item privacy is deleted.

This table also shows that there is one dimension for which the Cronbach’s α increases if an item is deleted. This applies to the item ‘Privacy’: if this item is deleted from the dimension *Autonomy*, Cronbach’s α will increase from 0,816 to 0,827. All the other items are necessary components of the dimensions assessed.

We also found a ceiling effect in our data: the percentage of patients giving the highest possible score is much higher (range 11,6% to 30,4%) than the percentage of patients who gave the worst possible score (range 0,1% to 1,0%). The item discriminant validity (IDV) shows that all items correlate more highly with the dimension they fit in than with the other dimensions (range 0,342 to 0,729).

The correlation of the dimensions with the other dimensions and with the overall satisfaction is given in Table 4.

**Table 4:** **Inter-dimensional correlations*Pre-admission visit (PAV), Admission (AD), Operation Room (OR), Nursing care (NC), Medical care (MC), Information (INFO), Autonomy (AUT), Discharge (DCH)*and correlation with overall satisfaction score**

**Dimensions COPS-D**	**PAV**	**AD**	**OR**	**NC**	**MC**	**INFO**	**AUT**	**DCH**	**Overall rate**
**PAV**	-	-	0,596**	0,650**	0,540**	0,633**	0,589**	0,628**	0.545**
**AD**	-	-	-	0,703**	0,477**	0,604**	-	-	0,547**
**OR**	0,596**	-	-	0,540**	0,620**	0,572**	0,529**	0,536**	0.527**
**NC**	0,650**	0,703**	0,540**	-	0,563**	0,658**	0,579**	0,615**	0,584**
**MC**	0,540**	0,477**	0,620**	0,563**	-	0,721**	0,618**	0,641**	0,558**
**INFO**	0,633**	0,604**	0,572**	0,658**	0,721**	-	0,702**	0,762**	0,623**
**AUT**	0,589**	-	0,529**	0,579**	0,618**	0,702**	-	0,701**	0,549**
**DCH**	0,628**	-	0,536**	0,615**	0,641**	0,762**	0,701**	-	0,573**

** sig 0.01.

The Spearman ρ-correlation with the overall satisfaction was significant at the 0.01 level for all dimensions. Also, all correlations could be considered as strong (>0,5) (range 0.527 – 0.623). The dimension *Information* correlated most strongly with patient ratings of overall satisfaction (ρ = 0,623). The inter-dimension correlation was also significant at the 0.01 level for all dimensions. All correlations except one (0.477) could be considered as strong (range 0.529 – 0.762). The strongest inter-dimensional correlation is the one between the dimensions *Information* and *Discharge*, the weakest correlation is the one between *Admission* and *Medical care*.

### External validity and known group differences

The results concerning the external validity and known group differences of the COPS-D are given in Table 5 and 6. Table 5 shows that our sample consists of less children than the LMR-data. Gender and age appeared to be comparable to the total Dutch day care population.

**Table 5:** External validity comparison dataset COPS-D with LMR-dataset regarding gender and age

**Gender**	**LMR-dataset day care 2008 (percentage)**	COPS-D dataset (percentage) (n = 3208)*
	(n = 1.185.276)	(n = 3702)
Female	57%	53%
Male	43%	43%
**Age**	**(n = 1.185.276)**	**(n = 3764)**
Younger than 20 years	11%	6%
20-59 years	45%	47%
60 years or older	44%	46%

*Not all numbers add up to 3802, due to missing values.

**Table 6:** Known group differences, relating COPS-D score means (SD) according to gender, age, education and health status (n = 3802)

	**PAV**	**AD**	**OR**	**NC**	**MC**	**INFO**	**AUT**	**DCH**
**Gender**								
Male	4,05 (0,66)	4,20 (0,67)	4,19 (0,64)	4,12 (0,74)	4,10 (0,86)	3,88 (0,80)	3,86 (0,75)	3,76 (0,85)
Female	3,97 (0,67)	4,24 (0,65)	4,14 (0,70)	4,06 (0,80)	4,03 (0.88)	3,86 (0,79)	3,82 (0,75)	3,69 (0,87)
F (df = 1)	3,5	1,3	1,9	**5,2**	**5,1**	0,7	1,7	2,0
p value	0,061	0,250	0,166	**0,022**	**0,024**	0,386	0,225	0,158
**Age**								
< 20 years	3,52 (0,79)	4,02 (0,77)	3,82 (0,89)	3,80 (0,92)	4,05 (0,79)	3,80 (0,766)	3,75 (0,79)	3,67 (0,92)
20-59 years	3,91 (0,66)	4,18 (0,67)	4,09 (0,66)	4,00 (0,78)	3,92 (0,87)	3,76 (0,80)	3,79 (0,73)	3,62 (0,85)
> 60 years	4,12 (0,64)	4,30 (0,61)	4,26 (0,67)	4,22 (0,72)	4,21 (0,82)	3,99 (0,78)	3,90 (0,78)	3,85 (0,86)
F (df = 2)	22,7	15,8	14,0	53,1	45,5	32,2	4,3	10,9
p value	**<0,0001**	**<0,0001**	**<0,0001**	**<0,0001**	**<0,0001**	**<0,0001**	**0,013**	**<0,0001**
**Education**								
None/lower	4,07 (0,67)	4,28 (0,65)	4,22 (0,66)	4,16 (0,78)	4,18 (0,81)	3,98 (0,77)	3,88 (0,75)	3,83 (0,83)
Moderate	3,99 (0,65)	4,19 (0,65)	4,16 (0,66)	4,04 (0,75)	3,99 (0,85)	3,84 (0,78)	3,84 (0,73)	3,70 (0,86)
Higher	3,96 (0,68)	4,17 (0,67)	4,10 (0,72)	4,04 (0,80)	3,99 (0,91)	3,73 (0,85)	3,77 (0,81)	3,59 (0,89)
F (df = 2)	2,6	**4,5**	**3,0**	**9,6**	**18,3**	**20,8**	2,5	**7,0**
p value	0,074	**0,012**	**0,049**	**<0,0001**	**<0,0001**	**<0,0001**	0,08	**0,001**
**Health status**								
Bad/moderate	3,93 (0,74)	4,11 (0,68)	4,08 (0,74)	4,00 (0,83)	3,99 (0,88)	3,74 (0,83)	3,75 (0,77)	3,57 (0,94)
Good/very good/excellent	4,04 (0,63)	4,22 (0,68)	4,19 (0,66)	4,09 (0,75)	4,08 (0,83)	3,89 (0,78)	3,87 (0,75)	3,78 (0,83)
F (df = 1)	**5,4**	**6,1**	**7,0**	**9,3**	**7,1**	**19,3**	**6,9**	**13,5**
P value	**0,02**	**0,013**	**0,008**	**0,002**	**0,008**	**<0,0001**	**0,009**	**<0,0001**

Pre-admission visit (PAV), Admission (AD), Operation Room (OR), Nursing care (NC), Medical care (MC), Information (INFO), Autonomy (AUT), Discharge (DCH).

Table 6 shows significant differences regarding gender, age, education and health status. Comparison by gender showed significant differences for two dimensions, nursing care (male 4,12 (SD = 0,74), female 4,06 (SD = 0,80), p = 0,022) and medical care (male 4,10 (SD = 0,86), female 4,03 (SD = 0,88), p = 0,024). Older patients are more satisfied on all eight dimensions. Similarly, healthier patients were more satisfied consistently. Lower educated patients were more satisfied, except with the dimensions *Pre-admission visit* and *Autonomy*.

### Acceptability

One of the remaining 24 items of the COPS-D had 12% missing values: ‘Transfer of information to external professionals’. All the other items had less than 8% missing values (range 0% to 7,9%).

## Discussion

This paper describes the development and validation of the COPS-D for the measurement of patient satisfaction with day care in hospitals. Patient satisfaction is seen as an important indicator of quality of care [11-13,23,26-28]. This study meets the need of Dutch hospitals for a valid and reliable questionnaire for day care patients given the rising organisation of day care in recent years.

The day care questionnaire is based on the COPS, a well validated questionnaire to measure patient satisfaction for clinical and outpatient hospital care. It is adapted to the day care situation with two additional dimensions: *Pre-admission visit* and *Operation room*. Part of the day care patients will have surgery in the operation room and/or have an intake visit with a nurse. The importance of the pre-admission visit for day care is supported by the literature: it is associated with reduced anxiety and increased satisfaction [5,6,29-31]. The questionnaire was tested in a large sample of patients from five general hospitals in the Netherlands.

### Construct validity and reliability

Based on the correlations being higher than 0.7, we concluded that the items ‘Personal attention surgeon’, ‘Information surgeon’ and ‘Transfer of information’ in the COPS-D measure the same construct as items in the COPS. It is reasonable to assume that the surgeon is the only doctor that the patient sees during the day care admission. We therefore deleted these redundant items from the dimension *‘Operation room’.*

The factor analysis confirmed the relevance of the eight dimensions of the questionnaire. The extraction communalities were higher than the threshold, as were the MSA.

Cronbach’s α’s showed good internal consistency within the dimensions. The item internal consistency was higher than the threshold, as were the inter-item correlations. The item discriminant showed that all items correlated stronger with the dimension they fit in than with the other dimensions. The results of the item ‘Privacy’ were erratic. If this item is deleted from the dimension *Autonomy,* the Cronbach’s α slightly increases. One could argue that the item ‘Privacy’ is not that important for a one day visit to the hospital and that it therefore should be deleted from the questionnaire. However, this item has inter-item correlations higher than 0,45 (0,530 with ‘Self-sufficient’ and 0,557 with ‘Participation in treatment’), extraction communalities are higher than 0,45 (0,640) and the item internal consistency is also higher than 0,40 (0,589). Therefore we decided to keep this item in the questionnaire. It could be interesting to investigate the scores on this item after collecting data from more hospitals with different kinds of day care facilities.

Patients’ overall satisfaction showed strong correlations (Spearman’s ρ >0,5) with the eight dimensions. From the literature [32] we know that overall satisfaction rates most highly correlate with factors associated with patients interaction with the hospital staff. This study showed that the dimension with the highest correlation with overall satisfaction in day care was the dimension *Information*. These findings correspond for example with the results of patient satisfaction research on pre-assessment clinics, where the provision of information was also an important indicator of patient satisfaction [29,33], and in day care for neurological patients, where patient satisfaction was not related to a new diagnosis or treatment, but rather to the amount of information and emotional support during the day care stay [33]. Patient information has become crucial in health care because it is necessary to enable the patient to take part in medical decisions and the resulting care provision [34].

The items in the COPS-D with the strongest correlation with overall satisfaction were ‘Transfer of information between professionals’ (ρ = 0,58), ‘Reception at the day care department’ (ρ = 0,57) and ‘Information about further treatment’ (ρ = 0,57). Next in strenght were ‘Expertise’, ‘Attention’ and ‘Information of the nurses’ (ρ = 0,55) and ‘Doctors’ (ρ = 0,53).

Apparently, the procedural items are more important to day care patients than to clinical patients, for whom interaction and attention of the professionals were most strongly related with their overall satisfaction [32]. Literature supports the finding that exchange of information between health care professionals and patients is essential, also because of time constraints and limited patient contact [5]. This might be an interesting field of future research. Especially the consequences for organisation of and attitude of professionals in day care centres deserve attention.

### External validity and known group differences

Although our sample involves less children (age 0–19 year) than the total Dutch day care population, the results of the two groups are comparable regarding gender and age. Therefore we assume that the results can be extrapolated to Dutch day care patients in general.

We found, as expected, that older and healthier patients are more satisfied with respect to all dimensions. We also found patients with lower education levels are more satisfied, except for the dimensions *Pre-admission visit* and *Autonomy* and that gender does not have a significant effect on satisfaction scores on all dimensions. These findings are in line with the literature [12,22,23].

### Acceptability

Clearly missing values are to be avoided. Our analysis showed that one of the remaining 24 items in this questionnaire had over 10% missing values. There is no strict rule regarding the maximum number of missing values to be considered acceptable. The number of missing data may be affected by a number of factors: the nature of the variable, the specialty a patient visited or the patient’s treatment [24,25].

The item ‘Transfer of information to external professionals’ clearly is not applicable to all patients. Twelve per cent of the day care patients did not answer this item on the questionnaire. This might depend on the specialty or treatment of the patient. Twenty per cent of the dermatology patients did not answer this item, as well as up to 7% of the patients coming for ear nose throat-surgery.

During our pilot study it appeared that day care organisation varied widely between Dutch hospitals. Day care centres differed in name, organisation of the department, employees and (medical) treatments or operations. For example, there are day care centres for surgery, for radiotherapy, psychiatric treatment, dialysis or diagnostics. Therefore, we suggest that this item is only added to the questionnaire if transfer to external professionals is indeed applicable. If this item is deleted, the Cronbach’s α still will be good (0,82 rather than 0,86). Another possibility for future research is to add the answering category ‘Not applicable’, because this might be the reason a relatively high percentage of patients did not answer the question.

As patient satisfaction is seen as indicator of quality of care and satisfaction may depend on the type of hospitalisation, it is reasonable to assume there is a difference in satisfaction between different kinds of hospital care [32,35]. Day care patients receive a different kind of care than clinical patients. The logistics and atmosphere of day care departments are different in the inpatient clinic. In general, day care patients are in better health than clinical patients. Moreover, health status influences patient satisfaction [23,32,36], as do individual conditions, treatments and preferences [37]. It can be expected that day care patients are more satisfied with day care facilities than clinical patients with inpatient care [30,38,39]. This corresponds with findings about patient satisfaction about day care admission for neurological second opinions or tertiary referrals [33]. Because the Clinical COPS, Outpatient COPS and COPS-D contain several identical questions, a comparable study can identify possible differences between the three types of care. This is an interesting topic of future research.

### Limitations

A number of limitations of the study design must be mentioned.

First, we could not study the characteristics of the non-responders, because of anonymity. Although our response rate is reasonable [14,40], extremely (dis)satisfied patients may not have returned the questionnaire. However, former research showed that the impact of non-response bias on satisfaction questionnaires of hospitalized patients is relatively small [34,41]. Also the external validity results showed that although our sample involves somewhat less children than the population of day care patients in the Netherlands, the groups are comparable regarding gender and age. Therefore, we assume that our non-response bias is limited.

Secondly, although the COPS is entirely based on the needs of clinical patients [13], day care patients were not specifically involved in constructing the COPS-D. The adaption is based on suggestions of professionals in hospitals that provide day care. We have assumed that the six general dimensions of *Admission, Nursing care, Medical care, Information, Autonomy* and *Discharge and aftercare* were also important to day care patients. We indeed found high correlations between these dimensions and the day care patients’ overall satisfaction. Still, there might be other aspects of day care which are important but not yet covered in the COPS-D. In-depth interviews and focus groups could further establish the content validity of this questionnaire for day care patients.

One of the arguments against assessing patient satisfaction is the skewed score distribution found regularly [34,42]: most patients are satisfied with the care they receive and only very few are dissatisfied. We indeed found a ceiling effect in our data: the highest percentage of the maximum score given addresses medical care (given by 30,4% of the patients). Nevertheless, high satisfaction figures do not mean that there is no room for improvement [36]. In this study we also see differences in means between the dimensions: e.g., comparing the mean score for *Admission* (M = 4,22) and the mean score for *Discharge* (M = 3,72). Thus, there is still room for improvement.

## Conclusions

The COPS-D is a valid and reliable questionnaire for assessing day care satisfaction. It completes the model of measuring patient satisfaction of the common types of care given in hospitals. The added value when compared to the COPS consists of two new dimensions: *Operation room* and *Pre-admission visit*.

The COPS-D fulfils the conditions made in advance while developing the Clinical and Outpatient COPS: a short, core instrument to screen patient satisfaction. This important information about hospital performance can be used to plan quality improvements. Over the next years, it becomes important to investigate whether hospitals indeed base their quality improvement activities on these patient satisfaction measurements and to what extent patient satisfaction improves after implementing such quality improvement activities.

## Competing interests

There are no competing interests.

## Authors’ contributions

Concept and design (SK, TK, LZ, JH), collection of the data and literature (SK), statistical analysis and interpretation of the data (SK, LZ), drafting of the manuscript (SK, TK, LZ, JH), critical revision of the manuscript (TK, LZ, JH), supervision (JH). All authors read and approved the final manuscript.

## Appendix 1 COPS-D: Day care questionnaire

### Pre-admission visit

How satisfied were you with…

· the reception

· the personal attention of the nurse

· the expertise of the nurse

· the information and instruction

### Admission at the day care centre

How satisfied were you with…

· the reception at the day care centre

· the rapidity of being able to speak to by the staff

· the degree of support of the staff

### Operation room

How satisfied were you with…

· the reception at the Operation Room

· the personal attention of the operation staff

· the expertise of the operation staff

### Nursing care

How satisfied were you with…

· the personal attention of the nurses

· the expertise of the nursing staff

### Medical care

How satisfied were you with…

· the personal attention of the doctors

· the expertise of the doctors

### Information

How satisfied were you with…

· the clarity of information given by nurses

· the clarity of information given by doctors

· the way information was transferred from one person to another

· the rapidity of learning research results

### Autonomy

How satisfied were you with…

· the degree of encouragement to be self-sufficient

· the degree to which you could participate in treatment decisions

· the privacy you were given such as in conversations with doctors during physical examinations and during visiting times?

### Discharge and aftercare

How satisfied were you with…

· the information provided about further treatment

· the transfer of information to external professionals, such as your G.P.

· the discharge procedure

## Pre-publication history

The pre-publication history for this paper can be accessed here:

http://www.biomedcentral.com/1472-6963/12/125/prepub
